# Tongue Necrosis as an Initial Manifestation of Giant Cell Arteritis: Case Report and Review of the Literature

**DOI:** 10.1155/2015/901795

**Published:** 2015-01-31

**Authors:** Jose R. Zaragoza, Natalia Vernon, Gisoo Ghaffari

**Affiliations:** Division of Allergy & Immunology, Penn State Hershey Allergy, Asthma & Immunology, 500 University Drive, UPC II, Suite 1300, Hershey, PA 17033, USA

## Abstract

Giant cell arteritis (GCA) is a systemic vasculitis of medium and large arteries that mainly affects the external carotid artery. It is a diagnosis of the elderly that typically presents as low-grade fever, temporal tenderness, claudication of the jaw, and in some patients vision loss. In cases where GCA presents with atypical manifestations, the diagnosis may be more difficult, causing a delay in both diagnosis and treatment and ultimately leading to irreversible complications. In this paper, we present an atypical presentation of GCA with symptoms of neck swelling and lingual pain in an elderly female. These symptoms progressed to bilateral necrosis and eventual dislodgement of the tongue. Lingual necrosis is a severe potential complication in GCA. In patients presenting with lingual swelling, pain, and discoloration, GCA should be suspected and prompt therapy should be initiated to avoid irreversible complications.

## 1. Introduction

Giant cell arteritis is a chronic vasculitis of large and medium sized vessels that commonly affects those above the age of 50 years. It has an estimated incidence of 20 cases per 100,000 individuals and a prevalence of 1 in 500 individuals [1]. Scandinavian and North American females are most commonly affected. Clinical presentation is usually fever, claudication of the jaw, and temporal headache. Less common presenting symptoms include dysphagia, cough, hearing loss, and necrosis of the tongue [2].

The diagnosis of GCA is clinicopathological for which the American College of Rheumatology (ACR) has established classification criteria. To meet the diagnosis criteria, patients should present at least 3 out of 5 positive findings. The ACR diagnostic tool does not include any lingual symptomatology amongst its criteria [3, 4] (Table 1).

Lingual necrosis is a known complication of GCA that commonly affects one side of the tongue [5–8]. In a few cases bilateral necrosis has been described and is considered extremely rare due to the rich lingual vascular supply [2, 5, 9–11]. This paper presents a case of lingual pain and bilateral tongue necrosis secondary to GCA along with a literature review of this rare complication.

## 2. Case Report

A 68-year-old female patient was initially evaluated by her primary care physician for a nonspecific moderate headache and swelling of the neck for four weeks. She was treated with a course of antibiotics and nonsteroidal anti-inflammatory medications for ten days. After completing this therapy, the patient presented to the Emergency Department for acute onset of severe pain and progressive swelling of the tongue that was compromising her speech and deglutition. She was admitted to the hospital and treated with empiric antibiotic therapy and intermittent doses of systemic glucocorticoids for suspected sialadenitis. Progressive swelling of the tongue compromised feeding so the patient was then transferred to a tertiary care institution for further treatment and consulted with the allergy service for evaluation of angioedema as possible culprit on swelling.

**Figure 1:**
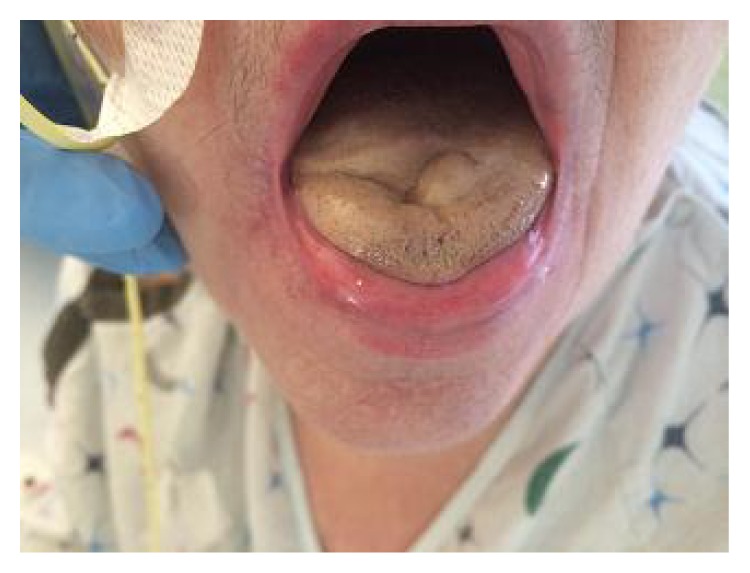
Discoloration and swelling of the anterior half of the tongue upon initial evaluation.

Patient denied symptoms of fever, malaise, or weight loss. She did not have vision loss, jaw claudication, or temporal tenderness. Past medical history was negative for vasoconstrictor use, radiation therapy, cardiac arrest, or embolization. Vital signs were within normal limits with no evidence of cardiac or pulmonary abnormalities. Edema and discoloration involving the anterior half of the tongue with movement limitation were the main findings on physical examination. No temporal artery tenderness was noted (Figure 1).

Hematologic testing revealed mild leukocytosis, anemia and thrombocytosis (WBC: 13.8 ku/L, Hgb: 12.4 g/dL, Hct: 37.8%, Plt: 414 ku/L), and mild neutrophilia on differential. The comprehensive metabolic panel was unremarkable.

Elevation in both C-reactive protein (CRP) and erythrocyte sedimentation (ESR) (1.3 mg/dL and 55 mm/hr, resp.) was concordant with an acute inflammatory process. Antineutrophil cytoplasmic antibody was negative. Even though her presentation was atypical for angioedema, serum C4 levels and C1 esterase inhibitor and function were measured and found to be normal.

A tongue biopsy was performed and showed coagulative necrosis, and no malignant cells or amyloid deposits were identified. Imaging studies including a CT-angiography showed luminal irregularity of right cervical internal carotid artery described to have a “beaded appearance,” suggesting arterial involvement and segmental luminal narrowing. A soft tissue defect of the anterior part of the tongue, consistent with avascular necrosis of the tongue, was noted.

**Figure 2:**
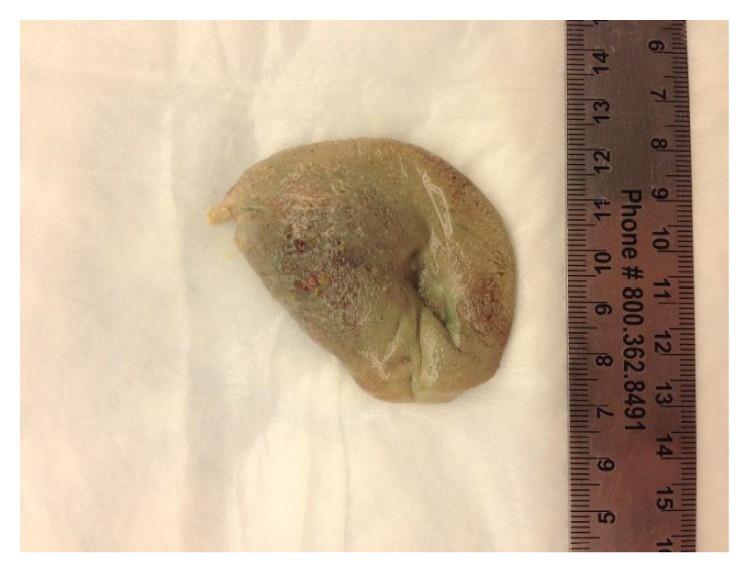
Necrotic appearance of the tongue posterior to dislodgement.

**Figure 3:**
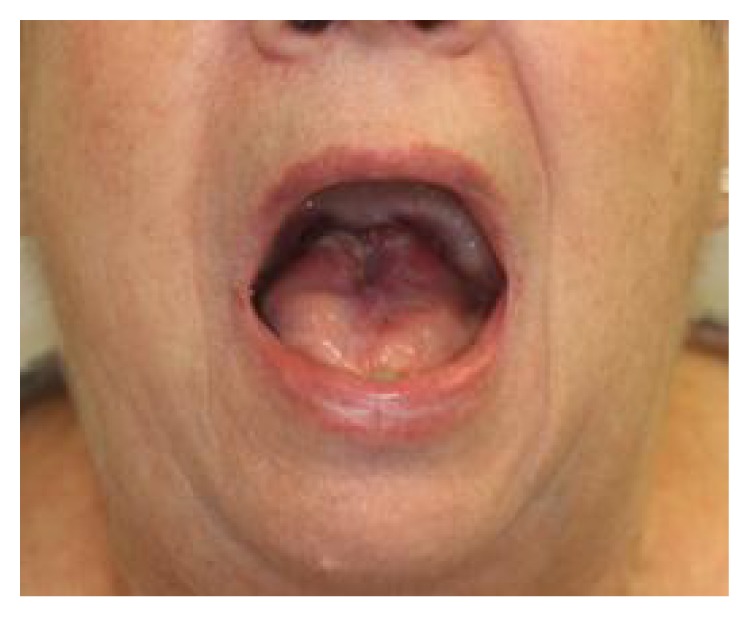
Appearance of the healed tongue.

GCA was confirmed with bilateral biopsies of the temporal arteries, which showed mixed inflammatory infiltrate composed of lymphocyte, histiocyte, and multinucleated giant cells with focal destruction of internal elastic lamina and marked intimal thickening significantly decreasing the lumen of both arteries, consistent with GCA vasculitis. Our patient met 3 of the 5 ACR criteria: age older than 50, elevated ESR, and histology consistent with GCA on both temporal arteries. Treatment with high dose corticosteroids was initiated at 1 mg/kg. Unfortunately, the patient had extensive necrosis of the tongue that progressed to self-amputation of the anterior half of the tongue (Figures 2 and 3). No other organ system was involved.

## 3. Literature Review

A PubMed based search was conducted combining the terms “giant cell arteritis and bilateral tongue necrosis,” “lingual necrosis,” and “ischemia.” It included articles published in English and Spanish. We found that bilateral tongue necrosis in GCA has been previously reported in 5 patients. A history of atypical symptoms like dysphagia, lingual pain, and swelling was present in all the cases described. Typical symptoms were only seen in one patient described by Zadic et al., who presented with pain on right side of the head, visual blurring, and fatigue before evaluation (Table 3).

## 4. Discussion

In general, necrosis of the tongue is rare and may be caused by a wide array of different pathologies ranging from lingual malignancy, use of vasoconstrictor, and radiation therapy [12–17] (Table 2). This paper presents a case of bilateral tongue necrosis and eventual self-amputation of the tongue as a rare complication of GCA. Our case and the literature review of patients with this rare complication reveal that these patients often present with lingual pain and the absence of the typical symptoms of GCA ultimately delaying diagnosis and treatment. Patients with lingual pain or swelling nonresponsive to treatment for infection or angioedema should raise a high index of suspicion for a vasculitic process such as GCA, especially in patients above the age of 50. Physicians should be familiarized with this presentation since lingual swelling may prompt a consultation for evaluation for suspected angioedema, as was the case in our patient.

## Figures and Tables

**Table 1:** Three out of five criteria are required to make the diagnosis of GCA.

American College of Rheumatology diagnostic criteria	
(1) Age older than 50 years at onset of disease.	
(2) New onset of localized headache.	
(3) Abnormal temporal artery with tenderness or decreased pulse.	
(4) Erythrocyte sedimentation rate higher than 50 mm/hr.	
(5) A biopsy of the artery showing necrotizing arteritis with predominant mononuclear cell infiltrate or granulomatous process with multinucleated giant cells.	

**Table 2:** Differential diagnosis in lingual necrosis.

Differential diagnosis in lingual necrosis	
(1) Malignancy: (carcinoma, lymphoma, and sarcoma)	
(2) Drugs: (vasopressin, chemotherapy, and ergotamine)	
(3) Radiation therapy	
(4) Cardiovascular: (hemorrhage, embolism, and cardiac arrest)	
(5) Infection: (syphilis, tuberculosis)	
(6) Systemic vasculitis: (giant cell arteritis, ANCA positive vasculitis)	

**Table 3:** Literature review of bilateral necrosis of the tongue in giant cell arteritis.

Reference	Patient age	Previous symptoms	Presentation	Studies	ACR criteria	Treatment
Oliver et al. [2]	79	An episode of collapse, decreased vision on left eye, and abdominal pain with segmental small bowel necrosis	New-onset, spontaneous bilateral necrosis of the tongue	Erythrocyte sedimentation rate (ESR): 78 Ultrasound of temporal artery: thickened artery with halo	Yes	Prednisolone and azathioprine 100 mg/d each

Zadik et al. [5]	78	Pain of the right head, neck, and shoulder. Fatigue, visual blurring, and weight loss for last 2 mo	Sore tongue, bilateral swelling and pain	Erythrocyte sedimentation rate (ESR): 69 Ultrasound of left temporal artery revealed occlusion	Yes	Prednisone 60 mg/d

Schurr et al. [9]	66	Slurred speech, dysphagia for 2 weeks	Swelling of the tongue with a greyish-purple color	Erythrocyte sedimentation rate (ESR): 120 CT angio.: 3 cm long stenosis with 50% at left common carotid artery	No	Prednisone 500 mg/d × 3 d, then 100 mg/d

Sainuddin and Saeed [10]	82		Acute abdominal pain, fever, and intestinal infarction	Autopsy confirmed giant cell arteritis affecting intestine and temporal artery	Yes	

Patterson et al. [11]	88	Generalized weakness. Painful, swollen, and discolored tongue for 10 days	Ischemic tongue, tender to palpation, and decreased movement		Yes	Prednisone 40 mg daily
